# Radioactive Phosphorylation of Alcohols to Monitor Biocatalytic Diels-Alder Reactions

**DOI:** 10.1371/journal.pone.0021391

**Published:** 2011-06-22

**Authors:** Alexander Nierth, Andres Jäschke

**Affiliations:** Institute of Pharmacy and Molecular Biotechnology (IPMB), Heidelberg University, Heidelberg, Germany; Massachusetts Institute of Technology, United States of America

## Abstract

Nature has efficiently adopted phosphorylation for numerous biological key processes, spanning from cell signaling to energy storage and transmission. For the bioorganic chemist the number of possible ways to attach a single phosphate for radioactive labeling is surprisingly small. Here we describe a very simple and fast one-pot synthesis to phosphorylate an alcohol with phosphoric acid using trichloroacetonitrile as activating agent. Using this procedure, we efficiently attached the radioactive phosphorus isotope ^32^P to an anthracene diene, which is a substrate for the Diels-Alderase ribozyme—an RNA sequence that catalyzes the eponymous reaction. We used the ^32^P-substrate for the measurement of RNA-catalyzed reaction kinetics of several dye-labeled ribozyme variants for which precise optical activity determination (UV/vis, fluorescence) failed due to interference of the attached dyes. The reaction kinetics were analyzed by thin-layer chromatographic separation of the ^32^P-labeled reaction components and densitometric analysis of the substrate and product radioactivities, thereby allowing iterative optimization of the dye positions for future single-molecule studies. The phosphorylation strategy with trichloroacetonitrile may be applicable for labeling numerous other compounds that contain alcoholic hydroxyl groups.

## Introduction

Over the past 25 years, many radioactivity-based assays for the determination of enzymatic activities were replaced by optical assays involving absorbance, fluorescence, or chemoluminescence. At the same time, optical detection was also intensively applied in studies regarding the folding, dynamics, and interactions of biomolecules with other cellular components. Single-molecule FRET spectroscopy and super-resolution microscopy are just two very modern examples in which dye-labeled biomolecules play a central role [1], [2]. The covalent attachment of bulky and often hydrophobic dyes to a protein or nucleic acid may, however, dramatically influence its properties, and the function (e. g., catalytic activity) of such labeled constructs therefore needs to be thoroughly validated in order to collect meaningful data. This is not a trivial task, as (i) biomolecules with site-specifically attached dyes are precious and often availble in pico- or femtomole amounts only, and (ii) the attached dyes often interfere with the optical assays used for activity determination due to quenching, spectral overlap, or other phenomena. Therefore, there is a need for highly sensitive methods for enzymatic activity determination that are not disturbed by dyes, quenchers, or other modifications present in the biomolecule. We reasoned that radioisotopic tagging of the substrate of enzymatic reactions with ^32^P phosphate, followed by chromatographic analysis, should provide a solution to this problem.

**Figure 1:**
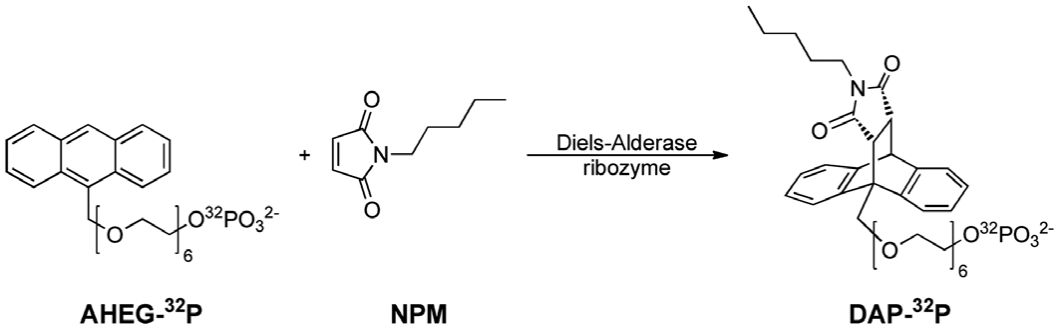
Conceptual approach to monitor the Diels-Alder reaction between the two substrates of the Diels-Alderase ribozyme. The ^32^P radioactively labeled anthracene-hexa(ethylene glycol) AHEG-^32^P and *N*-pentylmaleimide NPM yield the ^32^P-labeled Diels-Alder product DAP-^32^P. Substrate conversion and product formation were monitored by chromatographic separation and densitometric analysis of the spot intensities.

Our laboratory has discovered RNA enzymes that catalyze C-C bond formation by Diels-Alder reaction between two small organic (non-RNA) substrates [3], [4]. Since its discovery, the Diels-Alderase ribozyme (DAse) was studied by a multitude of chemical, biophysical and computational techniques as we ultimately aim to unravel the complex mutual relationship between folding, substrate binding and catalysis [5]–[10]. In our recent work we have used fluorescence-based single-molecule techniques to perform folding studies of the free ribozyme in solution [5]. However, finding the right label positions that do not disturb function for such a small and compact structure (49mer) turned out to be a challenging task, even with available crystallographic information. We observed precisely the problems outlined above: The attached dyes disturbed the readout of continuous fluorescence-and absorbance-based activity assays, and discontinous HPLC assays required prohibitive amounts of dye-labeled ribozyme. We therefore developed a general approach using radioactive phosphate labeling of the DAse standard substrate anthracene-hexa(ethylene glycol) (AHEG, Figure 1). In this model system, RNA-catalyzed kinetics between the phosphorylated diene and the (unlabeled) dienophile substrate *N*-pentylmaleimide (NPM) were recorded by thin-layer chromatographic separation of the reaction components and densitometric comparison of the substrate and product radioactivities.

The basic commercially available chemical for radioactive phosphorus nuclides is *ortho*-^32^phosphoric acid (H_3_^32^PO_4_). The ^32^P nuclide is a beta-emitter and gained its popularity in biochemical labeling due to its short half-life (14.3 days) and readily detectable energetic radiation (1.71 MeV). Usually, the specificity of enzymes is utilized to generate radioactive phosphate esters such as adenosine-5′-^32^phosphates (α-, β- or γ-ATP) [11], [12]. Since eligible enzymes like kinases or phosphatases are very specific in their substrate recognition [13], [14], a chemical synthesis procedure had to be developed to esterify the hydroxyl group of the AHEG substrate with radioactive phosphoric acid. The following requirements for the preparation of AHEG-^32^P had to be fulfilled: 1. A one-pot synthesis based on H_3_^32^PO_4_ as starting material. Other sources of ^32^P are difficult to obtain, dangerous, or reduce the specific radioactivity due to the preceding chemical conversions. 2. Facile purification to facilitate safe handling with radioactivity. 3. Appropriate yields and a H_3_^32^PO_4_/alcohol ratio close to unity to gain high specific radioactivity for the efficient detection of the Diels-Alder product – even in the presence of high excess of substrate. However, the synthesis of AHEG-^32^P meeting the aforementioned requirements turned out to be not a trivial task, since phosphoric acid is a rather unreactive substance and has to be activated first [15]. Most of the standard methods are hardly compatible with radioactive labeling, because several problems complicate the chemical phosphorylation, like the formation of multiple phosphates, di- or trialkylesters, and their laborious chromatographic separation.

Here we devised a simple and easily implemented synthetic route to ^32^P-phosphorylated alcohols and developed a TLC-based assay to accurately monitor the catalytic activities of dye-labeled ribozyme variants.

## Results

### Chemical phosphorylation of the alcoholic ribozyme substrate

To our surprise, adequate chemical methods to attach a phosphate to alcohols are scarce in the current literature. Phosphoryl chloride POCl_3_ in combination with a tertiary amine is the classic chemical agent. In principle, the radioactively doped derivative can be generated by the exchange reaction with PCl_5_ and inorganic ^32^P-phosphate, but the product ^32^POCl_3_ is volatile, dangerous and of low specific activity [16], [17]. Other procedures use phosphate activation as trimetaphosphate [18] or phosphoramidite [19], or employ secondary activation agents like cyanogen bromide [20], [21], imidazoles [22], or acetic anhydride [23].

**Figure 2:**
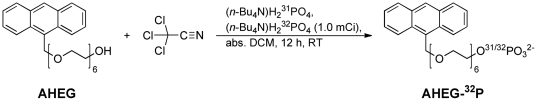
Synthesis of radioactively labeled anthracene AHEG-^32^P as ribozyme substrate. The alcohol of AHEG (1.0 eq.) was phosphorylated by activation with trichloroacetonitrile (1.3 eq.). The solubility of the phosphates in the organic solvent was achieved by using the tetrabutylammonium salt of phosphoric acid (1.3 eq.) which was doped with 1.0 mCi of *ortho*-^32^P-phosphoric acid prior to synthesis.

After unsatisfying phosphorylation attempts with some of these methods [21], [24], [25], we considered a more recent approach using organometallic synthesis [26]. The oxorhenium(VII)-catalyzed direct condensation of alcohols with phosphoric acid was, however, found to be inappropriate for the handling of radioactive compounds (reflux at temperatures above 140°C), and milder reaction conditions led to product mixtures [27] which were difficult to separate. Eventually, the synthesis of AHEG-^32^P succeeded by activation of the phosphoric acid with trichloroacetonitrile under anhydrous conditions (Figure 2). This reagent was first used by Cramer and co-workers in the early sixties for the synthesis of mono- and diphosphoric acid esters of the terpene alcohols, but was not developed further thereafter [28], [29].

Electron withdrawal by the three chlorine atoms facilitates nucleophilic attack of alcohols, forming intermediate trichloroacetimidates, which are versatile activated intermediates in synthetic chemistry [30]. In order to promote solubility of the phosphates in organic solvents, the tetrabutylammonium salt of non-radioactive phosphoric acid was employed, that was doped prior to use with 1.0 mCi of radioactive phosphoric acid. The removal of water from the aqueous phosphate stock solution and systematic control of pH were found to be crucial for the success of the reaction. The former was achieved by azeotropic coevaporation with acetonitrile and the latter by addition of tetrabutylammonium hydroxide to maintain pH 8. As this reaction was accompanied by side products, such as di- or triphosphates, we optimized the reaction conditions to obtain preferably the monophosphate ester of the alcohol. By varying the reactant ratios and reaction time, we achieved best results with 1.0 equivalent alcohol, 1.3 equivalents trichloroacetonitrile and 1.3 equivalents *ortho*-phosphoric acid for one hour at room temperature. The isolation of the AHEG-^32^P was achieved by preparative thin-layer chromatography (TLC) on silica-coated sheets using an ammonia-based mobile phase [31]. A sample of the purified AHEG-^32^P was analyzed by TLC, comparing of the intensity of the sample spot with the total radioactivity in the TLC lane, and purity was determined to be >96%. The yield of the labeling reaction was 50% and the specific radioactivity of the product was 51 mCi/mmol, which was convenient to perform precise kinetic measurements of RNA-catalyzed Diels-Alder reactions.

### Radioactive assay to measure catalytic activities of ribozymes

The addition of a charged phosphate at the end of the hexa(ethylene glycol) tether considerably improved water solubility and acceptance as substrate by the ribozyme (see Figure S1), thereby facilitating kinetic studies over a wide concentration range not accessible with other substrates [32], [33].

**Figure 3:**
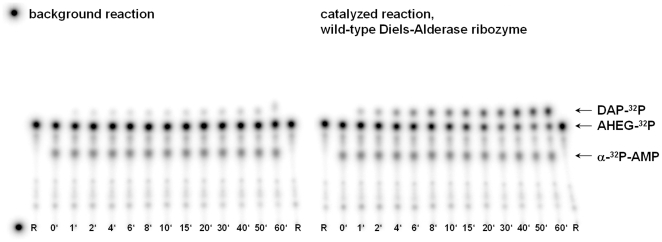
Radioactive assay for activity measurements of Diels-Alderase ribozymes. The picture shows thin-layer plates for the thermal background reaction (left) and the RNA catalyzed reaction (right) scanned on a Typhoon imager for radioactivity. For each point in time in minutes, one aliquot of the reaction mixture (0.7 µl) was spotted. Abbreviations are R = reference (AHEG-^32^P alone), DAP-^32^P = Diels-Alder product spots, AHEG-^32^P = starting material spots and α-^32^P-AMP = internal standard spots.

For the radiometric measurement of the ribozyme-catalyzed reaction, we developed a discontinuous assay with separation of the labeled reaction components by thin-layer chromatography. Experimentally, the procedure was to mix the RNA and the diene AHEG-^32^P in the reaction buffer, together with a small quantity of commercially available adenosine-5′-monophosphate (α-^32^P-AMP) as radioactive internal standard. The reaction was started by addition of the dienophile NPM. In appropriate time intervals 0.7 µl aliquots of the 10 µl reaction mixture were spotted onto a thin-layer plate and the reaction was stopped by immediate evaporation of the solvent with a blow-dryer. Afterwards, the components of the reaction were separated by developing the thin-layer plates with the mobile phase. A representative depiction of the thin-layer plates for the reaction catalyzed by the wild-type Diels-Alderase, and for the uncatalyzed background reaction (without RNA) is given in Figure 3. The increase of the Diels-Alder product intensity and the decrease in substrate intensity are clearly visible. A densitometric analysis was performed by quantifying the intensities for each starting material and product spot in relation to the total intensity of all spots. Every sample was further corrected for the experimental pipetting error by a correction factor, based on the fractional intensities of the internal standard (for further details see experimental section). Plotting the product concentration against the reaction time and fitting of monoexponential curves allowed for the calculation of initial velocities. Under the reaction conditions employed (7.0 µM wild-type ribozyme, 0.1 mM AHEG-^32^P, 0.5 mM NPM), we obtained a rate of product formation of 10.9 µM min^−1^ for the RNA catalyzed reaction and 0.1 µM min^−1^ for the background reaction, in accordance with previous findings [32].

### Catalytic activities of FRET-labeled Diels-Alderase ribozymes

While various assays are available for the kinetic investigation of unmodified ribozymes; including fluorescence- or absorbance-based assays [9], [33], they are less adequate for the precise kinetic analysis of ribozymes labeled with fluorescent dyes, as the dyes interfere with the optical readout. This is where the ^32^P-based assay provides unique features and gives access to the kinetic data.

**Figure 4:**
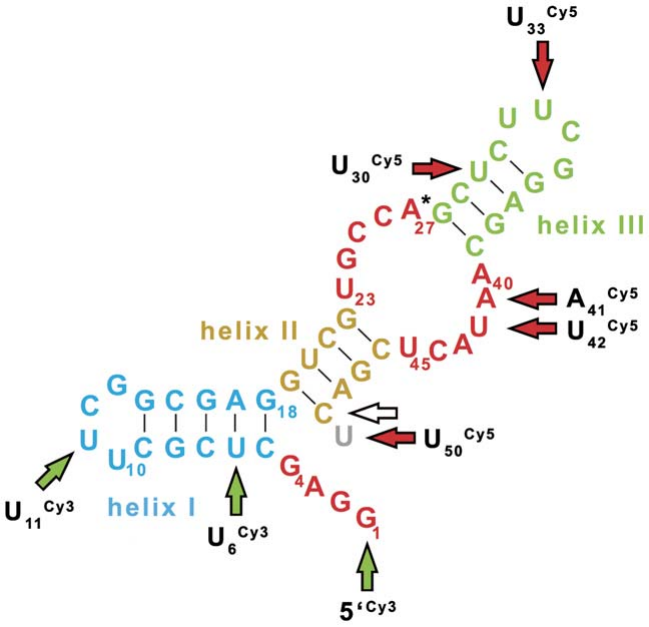
Depiction of 49mer Diels-Alderase ribozyme secondary structure with indication of all labeling positions. Green arrows refer to the Cy3 and red arrows to the Cy5 fluorophore. All constructs were 3′-biotinylated (hollow arrow) for immobilisation in future single-molecule experiments. In the case of 3′-Cy5-labeling, the 49mer minimal motive was extended to a 50mer by a dangling uridine (gray).

**Figure 5:**
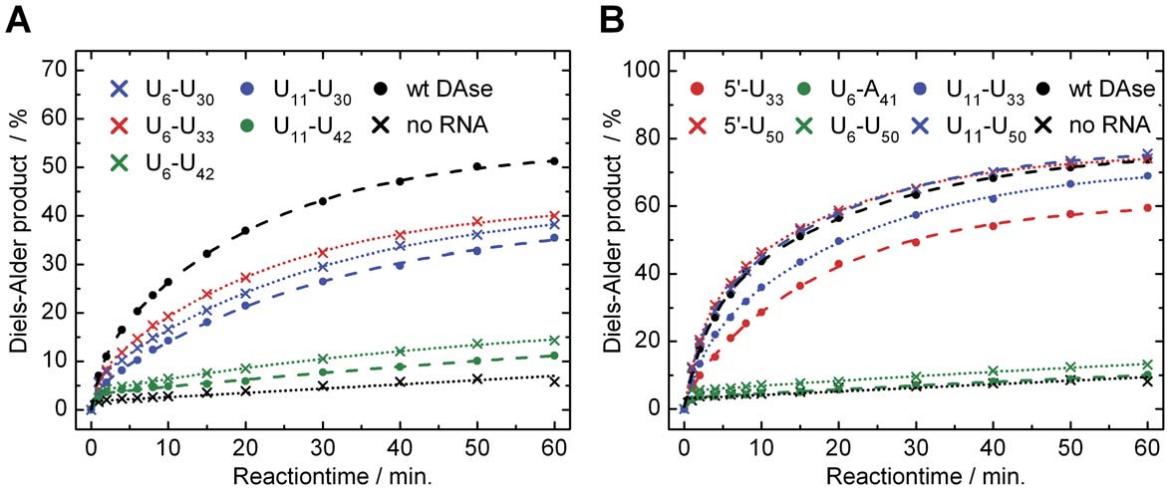
Reaction kinetics of dye-labeled Diels-Alderase ribozyme constructs determined by the radiometric assay. (A) Reaction kinetics of constructs with the first selection of dye-positions. Conditions: 7.0 µM RNA, 1.0 mM AHEG-^32^P and 1.0 mM NPM. (B) Reaction kinetics of constructs with optimized dye-positions, employing 0.1 mM AHEG-^32^P. References (black) are the reaction of the wild-type ribozyme (*wt* DAse) and the background reaction without RNA.

In the continuation of our previous single-molecule folding study of the ribozyme by Förster resonance energy transfer (FRET), we attempted to find improved label positions for the two cyanine dyes Cy3 and Cy5 [5]. By using the template-directed and combinatorial ligation strategy optimized in our lab [5], [10], we synthesized several RNA constructs with varying labeling positions (Figure 4). These constructs were then subjected to the radioactive assay, where readout was found to be as accurate as with the unlabeled ribozyme (see Figure S2). The first selection of dye positions resulted in RNA constructs of reduced catalytic activity, compared to the wild-type ribozyme (Figure 5A). Some constructs were almost inactive (both green traces), while the best construct showed four-fifths of the wild-type activity. Analysis of these results in the context of the available structural information [4] allowed to iteratively identify labeling positions with minimal perturbation of RNA catalysis. The kinetic curves of this “second generation” of constructs are shown in Figure 5B. As result, the constructs U_11_^Cy3^-U_50_^Cy5^ and 5′^Cy3^-U_50_^Cy5^ show wild-type and U_11_^Cy3^-U_33_^Cy5^ close to wild-type activity. Notably, labeling within the asymmetric bulge of the ribozyme leads to markedly reduced catalytic activities (constructs with labeling of A_41_^Cy5^ and U_42_^Cy5^).

## Discussion

The past popularity of radioisotopic labeling has been undermined by recent developments in fluorescence microscopy and spectroscopy, in which labeling with a fluorophore is an essential feature. For pure tracing of a biomolecular target, radioisotopic labeling has several advantages over organic fluorophores. Firstly radioactivity can easily be measured with high sensitivity down to the low femtomolar concentration range, and in terms of detection efficiency ^32^P is comparable or superior to the standard fluorophores. Secondly, the radiation of radioisotopes is completely independent of its molecular environment. This is in contrast to the modulation of fluorescence of organic fluorophores by proteins or nucleic acids which often leads to difficulties in data analysis and interpretation [34]. Furthermore, organic fluorophores are known to often interact with the 3D structure of the biomolecule and thereby interfere with their function. Such perturbations are much smaller or negligible for a phosphate group. Additionally, radiolabels do not suffer from self-quenching, photobleaching or signal fluctuations due to stacking and changes in pH or temperature.

An important advantage of radioactive labeling in our experimental setup is the independence from other fluorescence labels, *i.e.*, the dyes attached to the catalyst (“orthogonality”). This feature allows precise activity determination without interference. Furthermore, the radioactive assay presented here requires only minimum quantities of precious dye-labeled RNAs. Even with an inefficient enzyme (in terms of catalytic rate acceleration) like the Diels-Alderase, pmol amounts of enzyme are sufficient for reliable determination of the catalytic activity. The chemical labeling presented here avoids the need for enzyme-catalyzed transfer of phosphate and is therefore much wider in scope than most enzymatic approaches, which are often very restricted in terms of their substrate recognition. The one-pot synthesis using trichloroacetonitrile is experimentally easy to conduct, does not require demanding safety equipment, and can be performed in laboratories at standard radiosafety levels [17]. We expect that our methodology is adaptable to other enzymes that process substrates with an alcohol functionality or substrates to which an alcohol functional group can be appended. Notably, this methodology could prove useful for phosphorylation of other biologically or medically important alcohols [35], [36], and for the development of biochemical techniques that require ultrasensitive detection, like activity based protein profiling (ABPP) [37].

## Materials and Methods

### Materials and general procedures

Chemicals were purchased from Sigma-Aldrich (Taufkirchen, Germany) or Acros Organics (Geel, Belgium) and used without further purification. Radioactive compounds (H_3_^32^PO_4_, α-^32^P-AMP) were obtained from Hartmann Analytic (Braunschweig, Germany). The detection of radioactivity (^32^P nuclide) was performed with a β-γ handcounter (LB122 or LB124, Berthold Technologies, Bad Wildbad, Germany). High-sensitivity measurements of radioactivity were performed with a scintillation counter in the Cherenkov-mode (LS 650, Beckmann Coulter, Fullerton, USA). The specific radioactivity was calculated by comparing the radioactivity of the respective sample to the total radioactivity and by correcting for shielding effects. Standard DAse buffer (1×: 300 mM NaCl, 30 mM Tris-HCl, 80 mM MgCl_2_, pH 7.4) was prepared as 5× stock solution and diluted as required. RNA concentrations were measured in 2.0 µl sample volumes with a NanoDrop ND-1000 spectrophotometer (Peqlab Biotechnologie, Erlangen, Germany) using the theoretical extinction coefficient of the respective sequence at 260 nm (ε_260 nm_ = 453 600 M^−1^ cm^−1^). High-resolution electrospray ionisation mass spectra (HRMS-ESI) of phosphates were recorded on a Bruker micrOTOF-Q II in negative mode. Nucleic magnetic resonance (NMR) spectra were recorded on a Varian 500 MHz NMR system and calibrated using residual undeuterated solvent or d_6_-DMSO as internal standard. Visualisation and quantification of radiolabeled spots was conducted according to the “phosphorimaging” method: The thin-layer sheets were exposed to Eu^2+^-doped storage phosphor plates and readout was done with a Typhoon 9400 gel scanner (GE Healthcare, Uppsala, Sweden), equipped with a red solid-state laser (633 nm). The analysis of scans was performed with ImageQuant v5.2 software (GE Healthcare).

### Preparation of RNA

The (unlabeled) wild-type DAse was obtained from CSS Chemical Synthesis Services, (Craigavon, Northern Ireland). Ribonucleic acids with a FRET pair of fluorophores and a biotin modification were obtained by enzymatic ligation of separately modified 27mer (upstream) and 22mer (downstream) RNA fragments according to previously published procedures [5]. The eleven full length constructs were derived from the two sequences 5′-GGA GCU CGC UUC GGC GAG GUC GUG CCA-3′ (27mer) and 5′-GCU CUUCGG AGC AAU ACU CGA C(U)-biotin-3′ (22mer) carrying a Cy3 or Cy5 dye at one of the underlined bases. In some constructs, the 22mer was extended by one additional uridine (parenthesized) to incorporate a 3′-Cy5 dye. All RNA fragments and the 49mer splint DNA (5′-Fluorescein-GTC GAG TAT TGC TCC GAA GAG CTG GCA CGA CCT CGC CGA AGC GAG CTC C-3′) were obtained from IBA (Göttingen, Germany) as 100 µM stock-solutions and were used as received.

### Chemical Synthesis

(9-Anthracenylmethyl)hexaethyleneglycol (AHEG) [38] and *N*-pentylmaleimide (NPM) [39] were synthesized according to published procedures.

### Synthesis of (*n*-Bu_4_N)H_2_^31^PO_4_

In a 1.5 ml Eppendorf tube *n*-Bu_4_N(OH) (6.70 µl, 1.5 M in H_2_O, 10.05 µmol) and non-radioactive H_3_^31^PO_4_ (0.66 µl, 85% in H_2_O, 9.79 µmol) were mixed thoroughly with 100.0 µl acetonitrile (MeCN), frozen in liquid nitrogen and lyophilized to dryness.

### Synthesis of radioactively doped 1-(Anthracen-9-yl)-2,5,8,11,14,17-hexaoxa-nonadecan-19-yl phosphate (AHEG-^32^P)

The quarternary ammonium salt (*n*-Bu_4_N)H_2_^31^PO_4_ (1.3 eq., 9.79 µmol) was transferred into an Eppendorf vial with MeCN (25.0 µl). Then H_3_^32^PO_4_ (18.50 µl, 1.0 mCi, 37 MBq in H_2_O) was added and the solution was lyophilized to dryness. Alternatively, the solution was coevaporated twice with 50.0 µl of dry MeCN in a centrifugal evaporator. To the residue 50 µl dry dichloromethane and 3.5 µl dry AHEG (1.0 eq., 7.41 µmol) were added and the mixture was transferred to a fresh Eppendorf vial. The pH was checked by spotting 1.0 µl of the reaction mixture on a piece of pre-wetted pH paper (pH∼8) and the reaction was started by adding 1.01 µl trichloroacetonitrile (1.3 eq., 10.0 µmol). After gentle shaking of the vial in a thermomixer (Eppendorf Thermomixer comfort, Hamburg, Germany) for one hour at room temperature, the pH of the reaction was adjusted to pH∼8 by adding 1.5 µl *n*-Bu_4_N(OH) (1.5 M in H_2_O). The solution was concentrated in a centrifugal evaporator and washed three times with diethyl ether (500 µl). The aqueous residue was evaporated to dryness (30 min.), 35 µl dry dichloromethane, 3.5 µl dry AHEG (1.0 eq., 7.41 µmol) and 1.01 µl trichloroacetonitrile (1.3 eq., 10.0 µmol) were added and the solution was mixed over night. Afterwards, the solution was concentrated in a centrifugal evaporator, diluted with 20 µl H_2_O and the solution was washed three times with Et_2_O (300 µl, each ∼300 Bq/cm^2^).

The radioactive solution of AHEG-^32^P (∼80 µl) was applied on a thin-layer sheet (polyester supported silica, Polygram Sil G/UV_254_, 0.2 mm, 20.0×20.0 cm, Macherey-Nagel, Düren, Germany) with a positive-displacement pipette (100 µl, Pos-D, Rainin, Oakland, USA). Subsequently, the sheet was developed twice with freshly prepared running buffer [isobutyric acid/ethyl acetate/KCl (0.5 M in H_2_O)/NH_4_OH (25% in H_2_O), (7∶7∶2∶1)]. After short air-drying the product was located by UV fluorescence (R_f_ = 0.48) and the wet band was excised, collected in a 2.0 ml Eppendorf tube and extracted with MeCN/H_2_O (4∶3, 700 µl). After shaking for 15 min. the suspension was centrifuged (5 000 upm), filtered through a syringe filter (Teflon) and the solution was coevaprated three times with MeCN/H_2_O (2∶1, 1.5 ml) to dryness in a centrifugal evaporator. The residue was redissolved in water of the highest purity and the concentration was determined spectroscopically at 365 nm with a calibration line. For this purpose, thoroughly dried AHEG was weighed into vials and diluted to concentrations in the range of 20 to 150 µM, the anthracene absorption at 365 nm was measured and a linear fit of the data points yielded the equation:
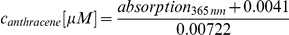



The purity of the product was determined by TLC, comparing the radioactivity of the compound spot with the total radioactivity of the chromatogram by densitometry. Yield was 50%, purity >96%, specific radioactivity 51 mCi/mmol. It should be noted that the specific radioactivity can be varied over several orders of magnitude by changing the ^31^P/^32^P ratio in the phosphorylation reaction. The purification of the product could also be performed by reversed phase HPLC (Agilent 1100 Series system) on a semi-preparative Phenomenex Luna C18 column (5 µm, 250×15 mm at 5.0 ml/minute). An isocratic mixture of the buffers A (0.1 M triethylammonium acetate in H_2_O, pH 7.4) and B (0.1 M triethylammonium acetate in MeCN/H_2_O, 5∶1, pH 7.4) was used for separation (A/B, 63∶37). Further analysis was conducted with the non-radioactive ^31^P-AHEG compound, which was purified by preparative TLC. ^1^H NMR (500 MHz, D_2_O/d_3_-MeCN, 25°C): δ = 8.68 (s, 1H), 8.46–8.48 (d, 2H), 8.17–8.19 (d, 2H), 7.67–7.71 (m, 2H), 7.60–7.64 (m, 2H), 5.63 (s, 2H), 3.98–4.00 (m, 2H), 3.86–3.89 (m, 2H), 3.77–3.79 (m, 2H), 3.40–3.70 (m, 15H), 3.22 (q, J = 7.3 Hz, 2H), 3.09 (dt, J = 7.2, 14.4 Hz, 1H) ppm; ^13^C NMR (126 MHz, D_2_O, 25°C): δ = 132.81, 132.19, 130.67, 130.43, 129.53, 128.49, 127.12, 125.67, 72.43, 71.48, 71.19–70.96, 66.33, 64.41, 48.23 ppm; ^31^P NMR (202 MHz, D_2_O, 25°C): δ = 1.01 (t, J(P,H) = 6.45 Hz) ppm. HRMS-ESI: m/z (%): calcd. for C_27_H_36_O_10_P: 551.2052 [M+H]^−^, found: 551.2071 (Figure S3).

### Radioactive Assay

Diels-Alder reaction kinetics (multiple-turnover) were measured by thin-layer chromatographic separation of the ^32^P-labeled reaction components and densitometric analysis of the substrate and product radioactivities. For a typical reaction, the RNA (final concentration: 7.0 µM), AHEG-^32^P (100.0 µM, ∼10·10^6^ cpm) and the internal standard α-^32^P-AMP (∼2.5·10^6^ cpm) were mixed in standard Diels-Alder buffer (1×) and the Diels-Alder reaction was started by adding and quick mixing of 1.0 µl of a 5.0 mM NPM stock solution (in dry EtOH containing 0.5% DMSO). The total volume of the reaction was 10.0 µl. The background reaction was measured by omitting the RNA. At different points in time aliquots were taken (0.7 µl), which were spotted on a TLC-plate (silica gel, Polygram SilG/UV_254_, 0.2 mm, 10.0×14.0 cm, Macherey-Nagel, Düren, Germany) and dried quickly with a blow-dryer for 30 s. After completion of one or several kinetic measurements, the TLC-sheets were developed twice with freshly prepared running buffer (see above). The radioactivity of the starting material alone (∼80 000 cpm) was set as reference point. Densitometric analysis of radioactivity was performed as follows: A correction factor for each sample was determined, by dividing the intensity of the respective internal standard spot α-^32^P-AMP (R_f_ = 0.34) by the average over all internal standard intensities in one experiment. Then the volume of each product spot (R_f_ = 0.55) and starting material spot (R_f_ = 0.47) was multipied with the respective correction factor. The amount of product formation was calculated by dividing the corrected product intensity by the sum of the corrected product and starting material intensities. Initial velocities were calculcated by fitting monoexponential curves to the datapoints with OriginPro v8.0 (OriginLab, Northampton, Massachusetts, USA).

## Supporting Information

Figure S1 **Catalytic performance of AHEG-^31^P in the absorbance based assay.** The measurement was performed at 365 nm. Conditions: 7.0 µM RNA, 0.1 mM anthracene derivatives and 0.5 mM NPM. The rate constant for non-radioactive AHEG-^31^P is equal to the fastest standard diene substrate 9-hydroxymethylanthracene (3.5 M^−1^ s^−1^) [33], as indicated by the near-identical progress curves for both reactions.
(TIF)

Figure S2 **Thin-layer plates from the reaction kinetics of dye-labeled Diels-Alderase ribozyme constructs, scanned for radioactivity (raw data).**
(PDF)

Figure S3 **Mass spectrum of non-radioactive AHEG-^31^P.**
(PDF)
