# Supplementary figures and images for: Radioactive Phosphorylation of Alcohols to Monitor Biocatalytic Diels-Alder Reactions
Source: PLoS One. 2011 Jun 22;6(6):e21391. doi: 10.1371/journal.pone.0021391 (PMC3120863; doi:10.1371/journal.pone.0021391)

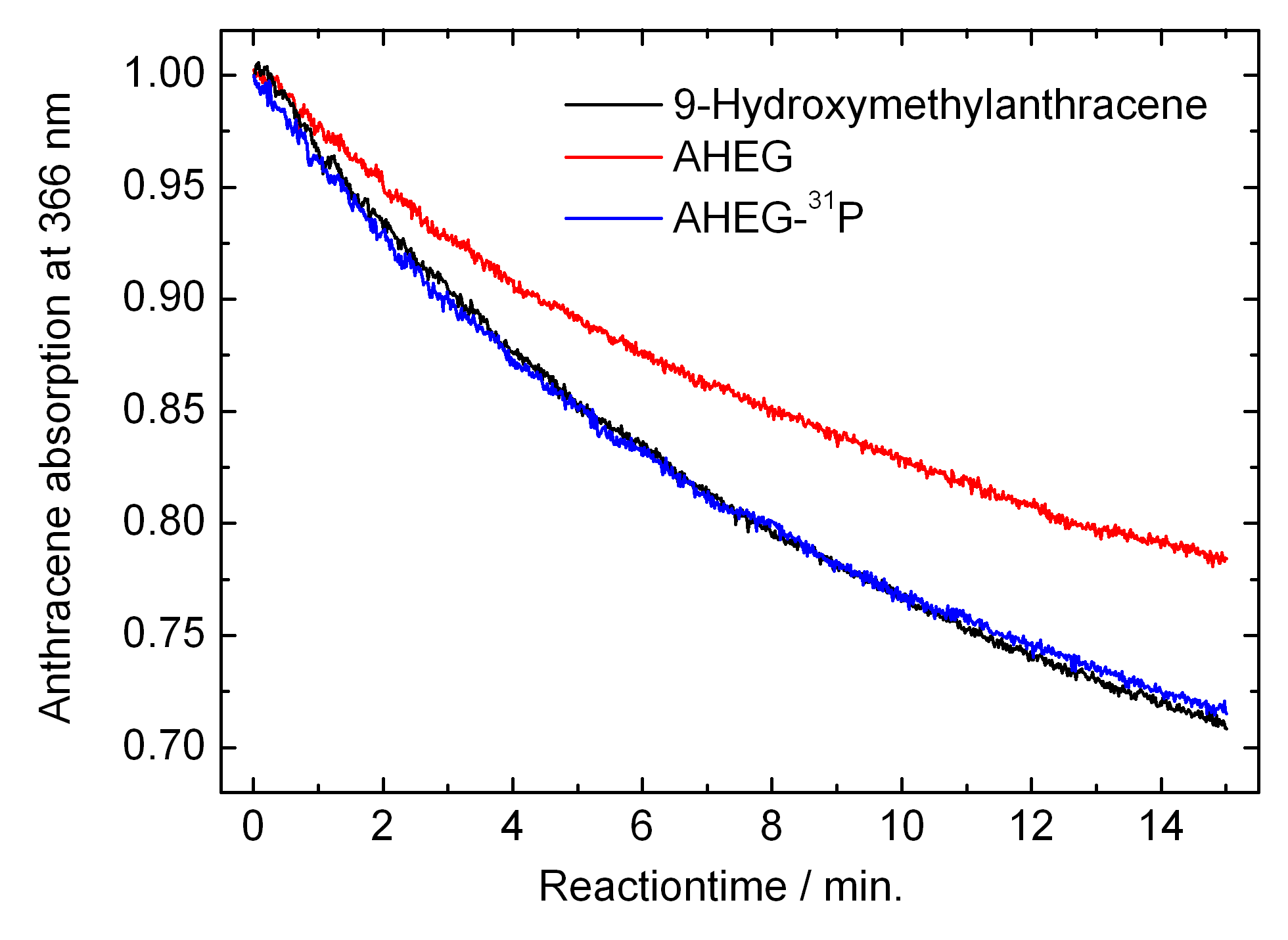

Supplement: Figure S1 — Catalytic performance of AHEG-31P in the absorbance based assay. The measurement was performed at 365 nm. Conditions: 7.0 µM RNA, 0.1 mM anthracene derivatives and 0.5 mM NPM. The rate constant for non-radioactive AHEG-31P is equal to the fastest standard diene substrate 9-hydroxymethylanthracene (3.5 M−1 s−1) [33], as indicated by the near-identical progress curves for both reactions. (TIF) [file pone.0021391.s001.tif]
