# Supplementary material for: Radioactive Phosphorylation of Alcohols to Monitor Biocatalytic Diels-Alder Reactions
Source: PLoS One. 2011 Jun 22;6(6):e21391. doi: 10.1371/journal.pone.0021391 (PMC3120863; doi:10.1371/journal.pone.0021391)

Dataset S1: Thin-layer plates from the reaction kinetics of dye-labeled Diels-Alderase ribozyme constructs, scanned for radioactivity (raw data)

U_6^{Cy3}-U_{30}^{Cy5}

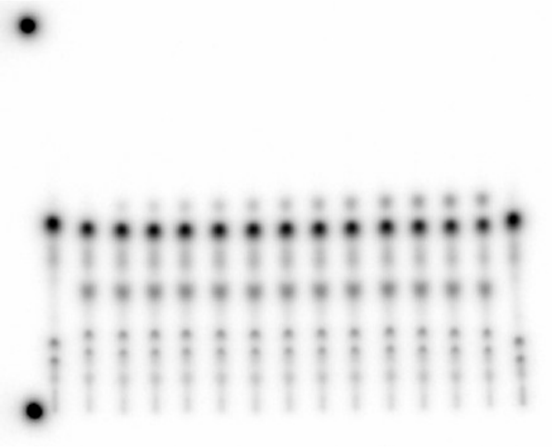

U_6^{Cy3}-U_{33}^{Cy5}

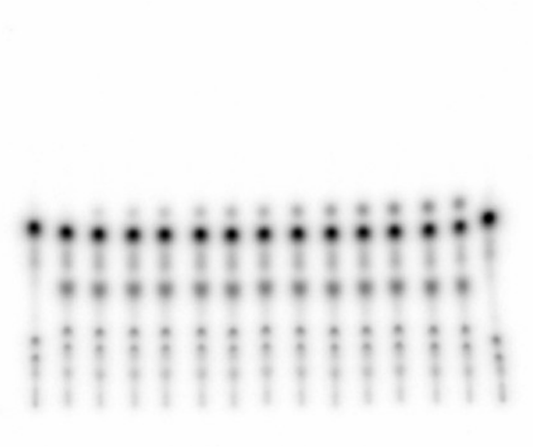

U_6^{Cy3}-U_{42}^{Cy5}

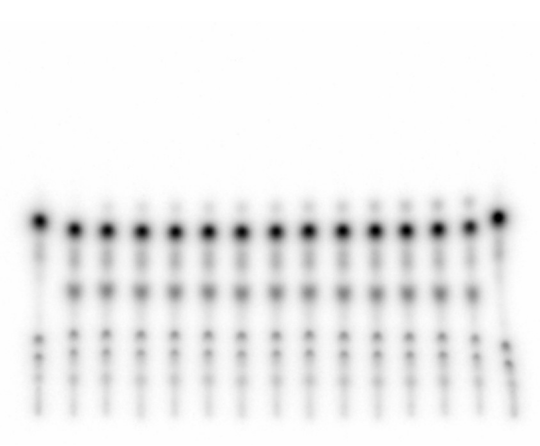

U_{11}^{Cy3}-U_{30}^{Cy5}

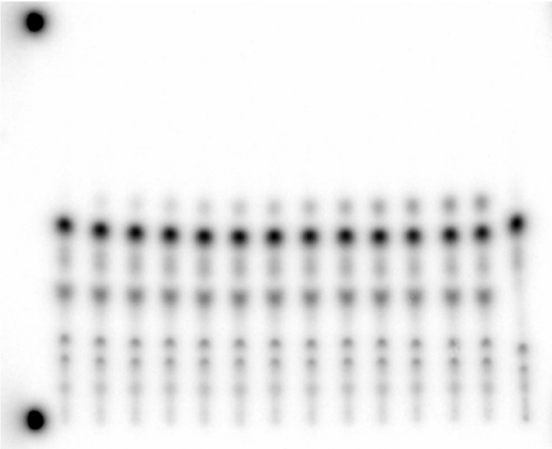

U_{11}^{Cy3}-U_{42}^{Cy5}

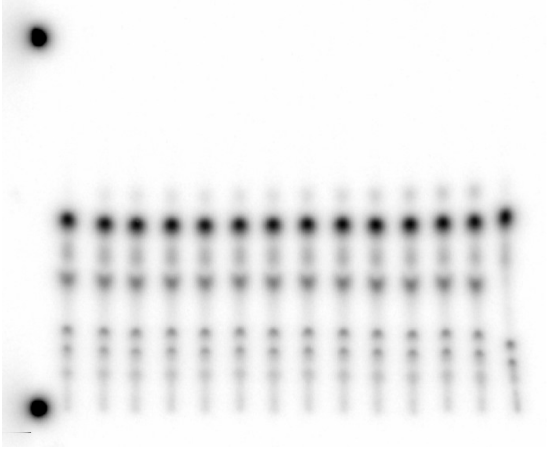

$5'^{Cy3}$-U_{33}^{Cy5}

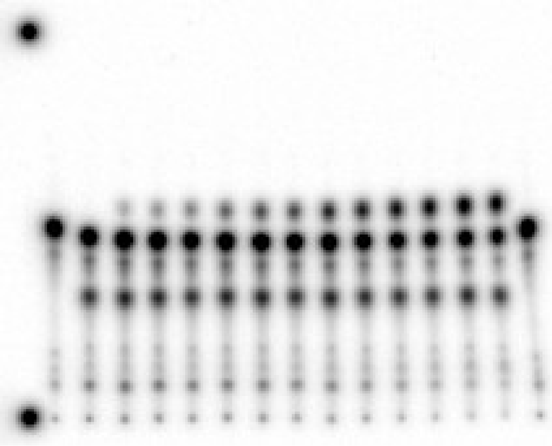

$5'^{Cy3}$-U_{50}^{Cy5}

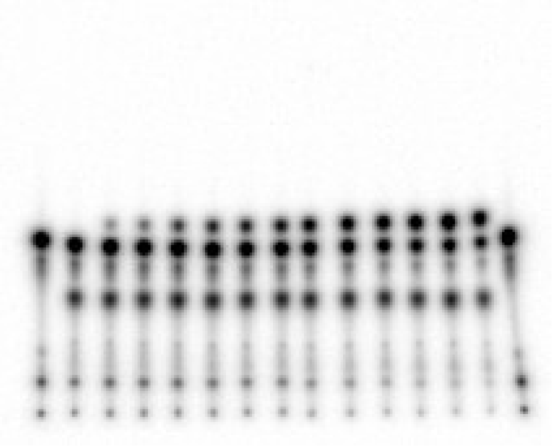

U_{6}^{Cy3}-A_{41}^{Cy5}

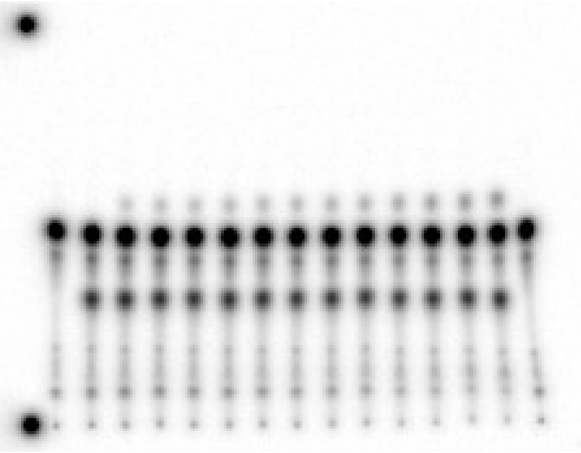

U_{6}^{Cy3}-U_{50}^{Cy5}

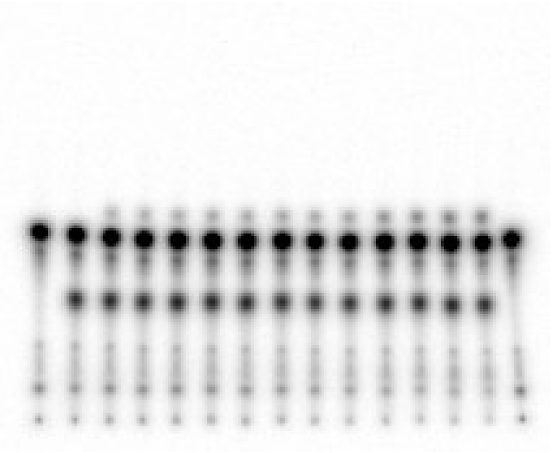

U_{11}^{Cy3}-U_{50}^{Cy5}

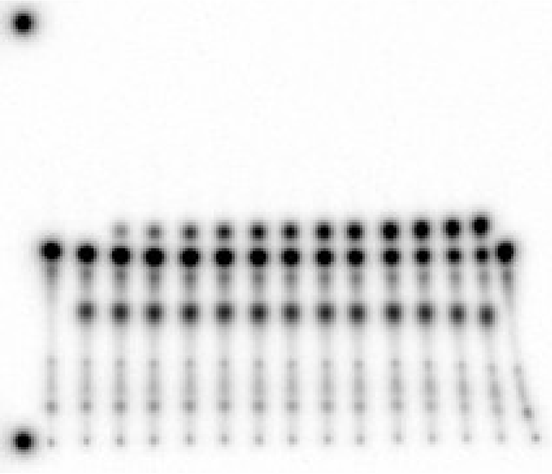

U_{11}^{Cy3}-U_{33}^{Cy5}

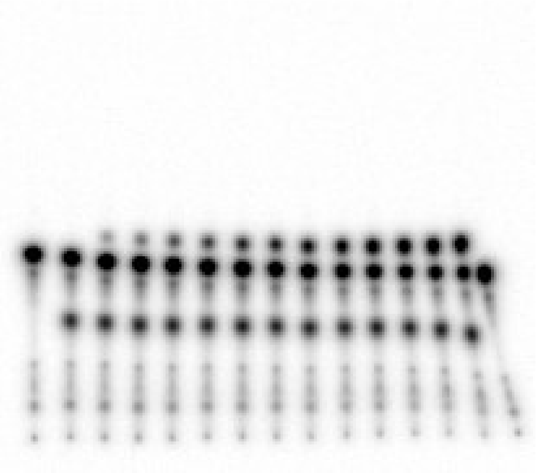

Supplement: Figure S2 — Thin-layer plates from the reaction kinetics of dye-labeled Diels-Alderase ribozyme constructs, scanned for radioactivity (raw data). (PDF) [file pone.0021391.s002.pdf]
