# Supplementary material for: Radioactive Phosphorylation of Alcohols to Monitor Biocatalytic Diels-Alder Reactions
Source: PLoS One. 2011 Jun 22;6(6):e21391. doi: 10.1371/journal.pone.0021391 (PMC3120863; doi:10.1371/journal.pone.0021391)

Dataset S2: Mass spectrum of non-radioactive AHEG-^{31}P

Display Report

Analysis Info

Analysis Name	W:\HEIKO\AN\III-ANI-129_B.d	Acquisition Date	08.02.2011 08:01:53
Method	HR_tune_low_neg_180-1200.m	Operator	Tof-user
Sample Name	C27H35O10P = 550,1979 g/mol	Instrument	micrOTOF-Q II 10254
Comment			

Acquisition Parameter

Source Type	ESI	Ion Polarity	Negative	Set Nebulizer	0.4 Bar
Focus	Not active	Set Capillary	2300 V	Set Dry Heater	180 °C
Scan Begin	50 m/z	Set End Plate Offset	-500 V	Set Dry Gas	4.0 l/min
Scan End	1200 m/z	Set Collision Cell RF	250.0 Vpp	Set Divert Valve	Source

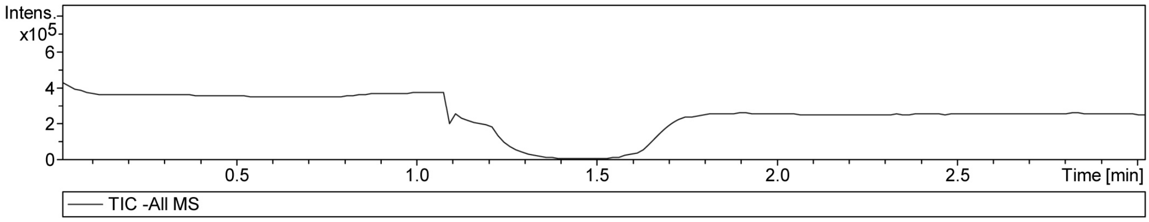

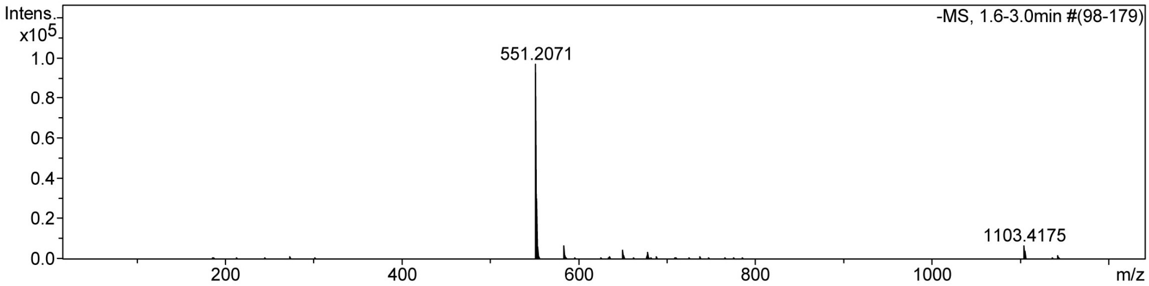

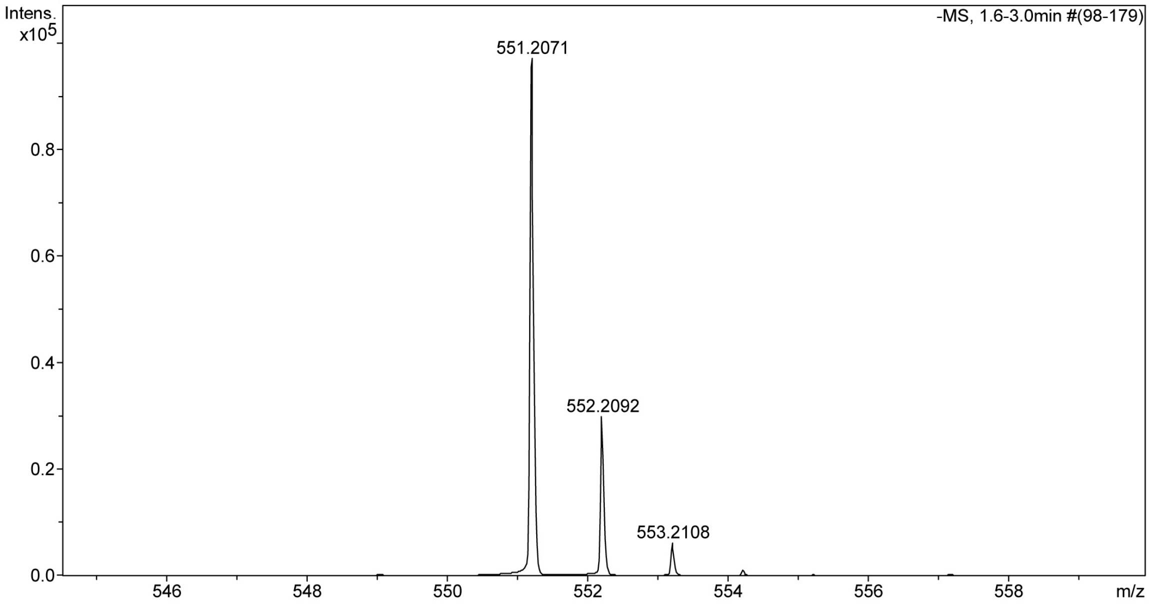

Supplement: Figure S3 — Mass spectrum of non-radioactive AHEG-31P. (PDF) [file pone.0021391.s003.pdf]
